# Phonon anomaly and local distortion in iron-manganese-based Elinvar alloys

**DOI:** 10.1080/14686996.2025.2540278

**Published:** 2025-08-04

**Authors:** Yoshihiko Umemoto, Yoichi Ikeda, Takashi Honda, Daisuke Ishikawa, John A. Schneeloch, Jörg C. Neuefeind, Shinichiro Tozawa, Rie Umetsu, Despina Louca, Alfred Q. R. Baron, Masaki Fujita

**Affiliations:** a Department of Physics, Graduate School of Science, Tohoku University, Sendai, Japan; b Institute for Materials Research, Tohoku University, Sendai, Japan; c Neutron Sciences Directorate, Oak Ridge National Laboratory, Oak Ridge, TN, USA; d Institute of Materials Structure Science, High Energy Accelerator Research Organization (KEK), Tsukuba-shi, Ibaraki, Japan; e Materials Dynamics Laboratory, RIKEN SPring-8 Center, Sayo-gun, Hyogo, Japan; f Department of Physics, University of Virginia, Charlottesville, VA, USA

**Keywords:** Iron-manganese based alloy, Elinvar, martensitic transformation, phonon softening, local distortion

## Abstract

This paper examines the phonon dispersion and static local atomic distortion of iron-manganese-based Elinvar alloys using high-resolution inelastic X-ray scattering, magnetization, neutron diffraction, and neutron total scattering. In this study, nonlinear phonon dispersion was observed for a transverse acoustic mode near zone center, associated with $(C_{11}-C_{12})/2$ elastic constants, over a wide temperature range along the Γ(220) to X (310) points of the face-centered cubic system, indicating lattice instability coupled with tetragonal distortions in the long-wavelength limit. Bulk magnetization and neutron diffraction measurements suggest that the conventional ferromagnetic magnetostriction scenario is not the origin of Elinvar characteristics. Instead, the martensitic transformation and lattice instabilities underlie these phenomena. The reduced pair distribution function reveals a significant discrepancy between local and global (averaged) structures suggesting the influence of atomic-scale lattice disorder and instability in FeMn-based Elinvar alloys.

## IMPACT STATEMENT

We identified local structures in iron-manganese alloys as a critical factor for understanding elastic properties at multiple scales and advancing the design of elastically invariable alloys, Elinvar, with enhanced versatility and efficiency.

## GRAPHICAL ABSTRACT

**Figure:**
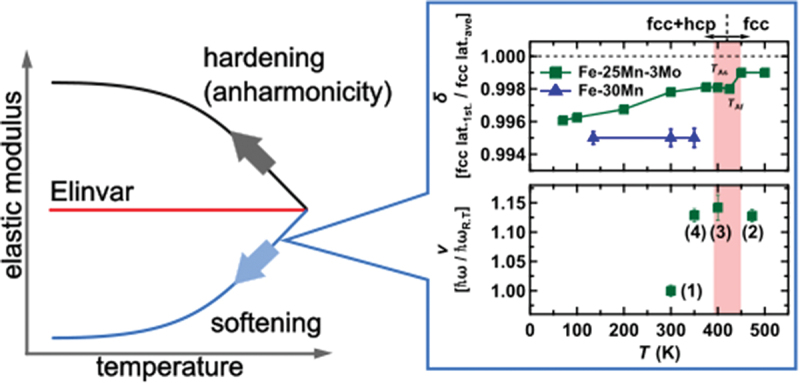

## 1. Introduction

**Figure 1.**
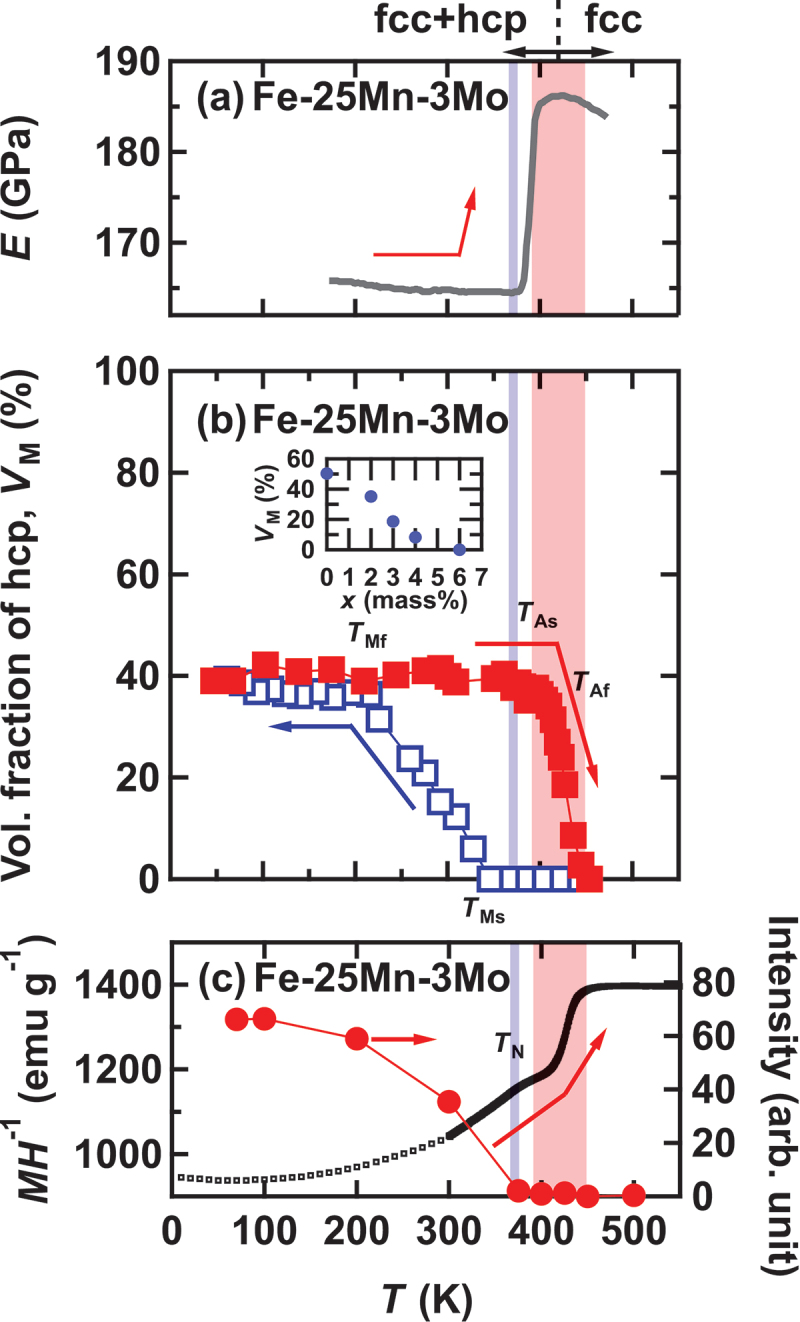
Temperature dependence of several parameters.(a) heating curve of Young’s modulus E. Reproduced by permission from [9]. (b) Volume fraction of the martensite (hcp) phase $V_{M}$. The filled red and open blue rectangles show results on warming and cooling, respectively. Inset in (b) shows the Mo concentration dependence of $V_{M}$ for Fe-25Mn-xMo at room temperature in cooling process. (c) Magnetization divided by $\mu_{0}H$ = 1 T. Low-temperature data, measured separately, are normalized at 300 K so that $MH^{-1}$ matches that of high-temperature data. In (c), the temperature evolution of the intensity of the magnetic Bragg peak measured by neutron scattering is also shown (red points, right axis). Solid lines serve as a visual guide. Data points are including error bar.

Invar and Elinvar characteristics have garnered attention in the field of fundamental solid-state physics and engineering science since C. E. Guillaume discovered prototypical invariable alloys at the end of the 19th century [1]. Subsequently, enhancing these invariable mechanical properties was crucial to improving the accuracy and stability of high-precision instruments, such as mechanical clocks, stress sensors, and seismometers. Consequently, various kinds of Invar/Elinvar alloys were developed in the 20th century [2–6]. Further in recent years, there has been interest in invariability with respect to magnetic field for the sensor applications [7]. Amidst these advancements, antiferromagnetic (AFM) or paramagnetic alloys have continued to receive attention, leading to the discovery of iron-manganese-based AFM Elinvar alloys by Masumoto et al. [8]. Illustrated by Figure 1(a), a ‘super’ Elinvar composition, AFM Fe-25Mn-3Mo (in mass percent), has an exceedingly small-temperature coefficient of Young’s modulus, dE/dT, at $-2.3\times 10^{-5}$ GPaK${\;}^{-1}$ across a wide temperature span of 253,373 K [8,9]. Furthermore, the FeMn-based Elinvars also show a good Invar characteristic in a similar temperature range [8,9]. This dual invariability, however, is challenging to explain with the conventional interpretation based on the ferromagnetic magnetostriction effect [10]. The challenge arises because uniform bulk magnetostriction does not occur in the AFM alloy, and the simultaneous appearance of Invar and Elinvar characteristics is not permissible, stemming from the different optimal points for spontaneous magnetostriction [11]. Additionally, paramagnetic Elinvar alloys, such as GUM METALL [12,13], require an advanced interpretation.

Considering the above issues, we investigate the influence of Martensitic transformation on the dual invariability, as we believe this may be an important additional factor. Indeed, the Elinvar characteristics for FeMn-based alloys emerge below the Austenite starting temperature, $T_{\mathrm{As}}$, where fcc and hcp mixed phase is shown in Figure 1 [8,9]. Accompanied by the structural transition, Young’s modulus E drastically increases toward $T_{\mathrm{As}}$. Above $T_{\mathrm{Af}}$, E decreases with increasing temperature like typical alloys due to anharmonicity. While elastic constant measurements below $T_{\mathrm{As}}$ would be effective in unveiling anomalous elastic behavior, they are difficult to interpret because of phase separation between Martensitic and parent phases complicates. To address this issue, reciprocal-space measurements have the potential to distinguish elastic properties between the two phases. In this paper, we present the results of high-resolution inelastic X-ray scattering, neutron diffraction, magnetization, and neutron total scattering measurements for FeMn-based Elinvar alloys, that, when taken together, suggest atomic disorder/instability, as related to the nearby martensitic transformation, play an important role in the Elinvar characteristics of the material.

## 2. Experimental procedure

### 2.1. Sample preparation and characterization

We prepared a series of polycrystalline Fe-yMn-xMo alloys using the arc melting method. The composition of arc-melted ingots was checked using a scanning electron microscope energy dispersive X-ray spectrometry (SEM-EDX). Its discrepancy from the target composition was within 2% in all samples as shown in Table 1.

**Table 1.** The sample compositions.

Sample	Target composition Fe:Mn:Mo (mass%)	SEM-EDX Fe:Mn:Mo (mass%)
Fe-25Mn	75:25	74.1:25.8
Fe-25Mn-2Mo	73:25:2	71.0:26.6:2.2
Fe-25Mn-3Mo	72:25:3	71.0:25.8:3.2
Fe-25Mn-4Mo	71:25:4	68.1:27.1:4.0
Fe-25Mn-6Mo	69:25:6	70.3:24.5:5.2
Fe-30Mn	70:30	69.8:30.0

The melted ingots were then cut into appropriate sizes using a high-speed cutter. All samples underwent annealing at 1173 K for 2 hours in an argon atmosphere to relieve mechanical stress, followed by cooling to room temperature in the furnace. These annealed samples were utilized for neutron diffraction, total scattering, and magnetization measurements. Additionally, we fabricated a rod-shaped sample measuring ϕ5.85 mm sample to assess the low-temperature elastic modulus using the electromagnetic ultrasonic resonance (EMAR) method. The elastic modulus of Fe-25Mn-3Mo was evaluated from the resonance frequency $f_{r}$ in the EMAR spectrum using $f_{r}\propto \sqrt{C/\rho }$, where C is an elastic constant and ρ is the density of sample. The resonance frequency increases with decreasing temperature below 300 K on both cooling and warming processes. The difference of elastic modulus was roughly 2% between low and high temperatures. The temperature coefficient of elastic modulus of our Fe-25Mn-3Mo sample is slightly larger than that of previous reports [8,9,14,15] while smaller than that of normal metals [16,17].

**Figure 2.**
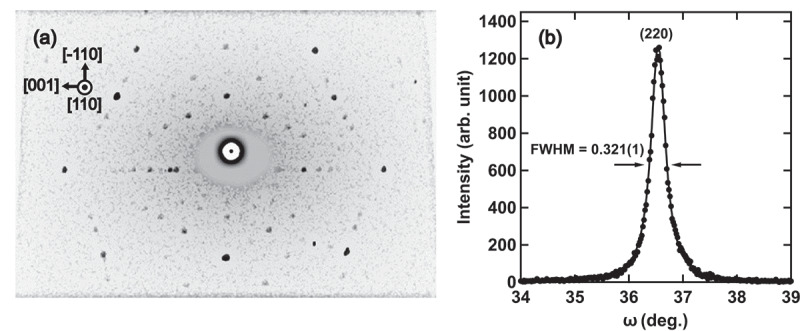
(a) Laue photograph of Fe-25Mn-3Mo for X-ray is approximately parallel to the ⟨110⟩. (b) The ω-scan profile of the single-crystalline Fe-25Mn-3Mo sample measured using Cu-Kα X-rays at the (220) Bragg position ($2\theta ∼74.24^{\circ }$). The solid line represents the result of a fit with the Lorentz function.

For inelastic X-ray scattering experiments, we grew a single crystal sample using a Bridgman method. A shaped polycrystalline ingot, pre-melted in an induction furnace at the Research Institute for Electromagnetic Materials, was placed into a Bridgman-type aluminum crucible, with a ceramic cap employed to minimize Mn evaporation, given its high vapor pressure. The vacuum chamber underwent multiple purges of high-purity argon at room temperature at around 300 K. Subsequently, the sample was heated in a carbon heater furnace to 1798 K, maintained for 1.5 hours to homogenize the melt, and then slowly cooled at a rate of 10 mmh${\;}^{-1}$ to 1603 K. Following this, the sample underwent further annealing at 1603 K for one week. Crystal axes were verified using a Laue diffractometer and Cu-Kα X-ray diffractometer, as depicted in Figure 2. A portion of the sample was then cut into approximately 3×3×2 mm${\;}^{3}$ using electrical discharge machining with the sample surface chosen to have a normal in the $(110{)}_{\mathrm{cubic}}$ direction. Following crystal axis verification, the surface was polished with #3000 sandpaper, and etched in aqueous FeCl${\;}_{3}$ for a few minutes. Lastly, the etched sample was annealed in an Ar atmosphere at 1173 K for 2 hours to further alleviate mechanical stress, before being slowly cooled to room temperature in the furnace.

### 2.2. Neutron diffraction

Neutron diffraction experiments were conducted using the HERMES neutron diffractometer installed at the T1–3 port of a thermal neutron guide tube in JRR-3 [18]. An Fe-25Mn-3Mo sample was sealed into a vanadium cell with a ${\;}^{4}$He exchange gas and mounted on a closed-cycle refrigerator. The bar-shaped sample measures approximately ϕ5.75 mm in diameter, with a length of around 60 mm and a weight of 7 g. In this experiment, the sample underwent a cooling cycle from room temperature to the base temperature of 30 K, followed by heating to approximately 480 K $\left(T>T_{Af}\right)$, and then cooling back to the base temperature through $T_{Ms}$. The incident neutrons were monochromatized using vertically focused Ge(331) crystals, and the neutron wavelength was calibrated to 0.2197 nm using a NIST standard 660c (LaB${\;}_{6}$). Data analysis was performed using the Fullprof suite package, and the volume fraction of the Martensitic and fcc-parent phases was evaluated via the Rietveld method [19,20].

### 2.3. Magnetization

Magnetization measurements were conducted using a vibrating sample magnetometer in the temperature range of 300 K to 900 K at 1 T, and a superconducting quantum interference device (SQUID) magnetometer in the temperature range of 10 K to 300 K at 0.1 T. The sample, weighing 0.4 g, was cut into a small rod-shaped ingot. Magnetization was measured during warming below 300 K and during both warming and cooling processes above 300 K. Following the high-temperature measurements, a slight ferromagnetic contamination was detected, likely stemming from the decomposition of an Fe-rich phase. Therefore, in this paper, we utilized only the data collected during warming below the decomposition temperature of 520–540 K, where magnetization exhibits a local minimum.

### 2.4. Inelastic X-ray scattering

Inelastic X-ray scattering (IXS) measurements were conducted using a high-resolution X-ray spectrometer at BL43LXU [21] of the RIKEN SPring-8 center. This beamline provides very high intensity with a large 2D array of analyzers allowing efficient measurements over a large area of momentum space [22]. Data were collected utilizing the Si (11 11 11) back reflection at 21.75 keV, with an effective energy resolution of approximately 1.4 meV, depending on analyzer. Following room temperature (∼300 K: $T_{Ms}<T<T_{As}$) measurements, the sample temperature was regulated using a closed-cycle cryo-furnace at 473 K ($T>T_{Af}$), 400 K, and 350 K ($T_{Ms}$) sequentially. Building upon prior work [23], we examined a transverse acoustic mode along the Γ(220) to X(310) point, which correlates with the shear elastic constant, $(C_{11}-C_{12})/2$.

### 2.5. Neutron total scattering

We conducted neutron total scattering experiments at BL21 NOVA [24] in MLF J-PARC and BL-1B NOMAD in SNS ORNL [25]. The measurements were carried out in the temperature range of 70 K to 500 K during warming. The bar-shaped samples, measuring approximately ϕ5.75 mm in diameter and ∼60 mm in length, were sealed into standard vanadium cells with a He exchange gas. Background contributions were measured at the same temperature and subsequently subtracted. Data corrected at NOVA were converted into the pair distribution function using Python scripts developed by the NOVA team [26], while those at NOMAD were converted using scripts developed specifically for NOMAD. All data were analyzed using PDFgui software [27] and fitted with simple random fcc/hcp structures to assess the degree of local lattice distortion [28].

## 3. Results and discussion

### 3.1. Characterization for Fe-25Mn-3Mo

First, we present the results of sample characterization. In Figure 1(b), the temperature dependence of the volume fraction of the Martensitic phase, $V_{M}$, for a super Elinvar composition Fe-25Mn-3Mo is depicted. These $V_{M}$ values were determined from the results of neutron diffraction experiments via Rietveld analysis. The red and blue points represent $V_{M}$ during the warming and cooling processes, respectively. The fraction of the Martensitic phase $V_{M}^{w}$ for the on-warming process is approximately 40% in the temperature range of 253–337 K, where Elinvar characteristics emerge. During the warming process (inverse transformation), the Martensitic phase begins to decrease above $T_{As}=$390(+5, −5) K and disappears entirely at $T_{Af}=$ 453(+0, −10) K. Here, the numbers in parentheses indicate the upper and lower limits of the uncertainty. Upon cooling above$T_{Mf}$, the Martensitic phase reappears below $T_{Ms}=$ 346(+0, −18) K, and volume fraction being saturated at $T_{Mf}=$ 215(+10, −0) K. Although $V_{M}^{c}$ evolves in the Elinvar temperature region during cooling, it reaches 40% below $T_{Mf}$. Consequently, $V_{M}^{RT}$ of Fe-25Mn-3Mo around room temperature can be estimated as approximately 15–40%. The characteristic temperatures for the Martensitic transformation are listed in Table 2.

**Table 2.** Characteristic temperatures for Fe-25Mn-3Mo evaluated from neutron diffraction and magnetization measurements. $T_{Ms}$ and $T_{Mf}$ represents the starting and finishing temperatures of the Martensitic and inverse Martensitic transformation respectively. $T_{N}$ represents the antiferromagnetic transition temperature.

	Starting temperature (K)	Finishing temperature (K)	AFM	$T_{N}(K)$
Martensitic	390 (+0, −18)	453 (+10, −0)	fcc(on warming)	377
Austenite	346 (+5, −5)	216 (+0, −10)	hcp	–

To assess the influence of Mo in the Fe-25Mn-xMo series, we investigated $V_{M}$ as a function of Mo concentration at 300 K. As shown in the inset of Figure 1(b), the $V_{M}$ is approximately 50% for the Fe-25Mn alloy ($x_{mass}=0$%), decreases with increasing Mo concentration, and disappears entirely above $x_{mass}∼6$%. These findings suggest that the ‘super-’ Elinvar characteristics emerge in a blended state of Martensitic and parent phases, indicating the potential presence of structural instabilities in both remnant fcc and transformed hcp phases. Specifically, the parent phase that remains untransformed during the cooling process retains structural instability over a broad temperature range, extending to low temperatures. In other words, within the mixed-phase region below the structural transformation temperature, the parent fcc phase, in particular, may exhibit significant strain energy, leading to anomalies in the elastic modulus via soft-mode phonons.

Next, we present the results of bulk magnetization measurements during the heating process. In Figure 1(c), we illustrate the temperature evolution of the magnetization $MH^{-1}$, where M is divided by the magnetic field $\mu_{0}H$ = 1 T. Below $T_{As}$, where fcc and hcp phases are still intermixed, $MH^{-1}$ exhibits a bend anomaly at $T_{N}^{fcc}∼$377 K due to an AFM transition in the parent fcc phase, as reported previously [8,9]. Our neutron experiments also observed magnetic Bragg peaks below $T_{N}^{fcc}$, as shown in Figure 1(c), consistent with the results of magnetization measurements. Between $T_{As}$ and $T_{Af}$,$MH^{-1}$ experiences a sharp increase, indicating a weaker magnetic state favored in the Martensitic hcp phase. $MH^{-1}$ remains nearly constant above the upper Martensitic transformation temperature $T_{Af}=$ 453 K, resembling a Pauli paramagnetic metal and the behavior of Mo-less FeMn alloys [29]. We note the following observations: (1) The Young’s modulus measured during warming does not exhibit any clear anomaly at $T_{N}^{fcc}$ [8,9,29,30]. (2) Below $T_{N}^{fcc}$, $MH^{-1}$ decreases monotonically, and no significant anomaly in $MH^{-1}$ is observed in the fcc – hcp mixed phase region. These findings suggest that the fcc antiferromagnetic state does not strongly affect the temperature dependence of the Young’s modulus.

Previous studies have reported magnetic transitions in the martensitic hcp phase of Fe–xMn alloys. In Fe–xMn alloys with low fcc-phase content (x=14,16,20), Mssbauer spectroscopy detected line broadening associated with the development of the hcp-AFM phase [31]. Moreover, in cold-worked Fe–28Mn, a weak and broad magnetic Bragg peak attributed to the hcp magnetic phase was observed [32]. On the other hand, in Fe–yMn–xMo alloys (y=20∼30,x=0∼6), no additional magnetic Bragg peaks were observed in the neutron diffraction profiles of the martensitic hcp phase below $T_{N}^{fcc}$ across the entire Mn concentration range. In general, magnetic Bragg peak intensity is proportional to the product of the magnetic phase volume and the square of the magnetic moment. Therefore, the hcp-AFM state appears to be destabilized as the volume fraction of the fcc phase increases. In hcp-rich or cold-worked samples, where the fcc phase is suppressed, the hcp magnetic phase tends to be stabilized. These results indicate that subtle differences in Mn concentration and strain state can significantly influence the magnetic properties of the hcp phase. The hcp phase volume fraction in our Fe–25Mn–3Mo sample is lower than that in previous studies [31,32]. We therefore consider that the hcp phase in our sample does not exhibit AFM ordering and does not affect the temperature dependence of the elastic properties.

### 3.2. Phonon dispersion anomaly in Fe-25Mn-3Mo

IXS measurements were conducted at 300, 473, 400, and 350 K in sequence. Considering the findings from sample characterization, the initial measurement at room temperature (300 K) is presumed to be conducted in the intermixed state of the Martensitic and parent fcc phases, where $V_{M}$ ranges from 15% to 40%. The temperature of the second measurement (473 K) is entirely above the upper Martensitic transformation temperature $T_{Af}=$ 453 K, placing the measurement within the parent fcc phase. The temperatures for the third and fourth measurements, both 400 K and 350 K, are higher than $T_{\mathrm{Ms}}$. At these temperatures, the on-cooling Martensitic transformation has yet to occur, and the parent fcc phase remains intact.

**Figure 3.**
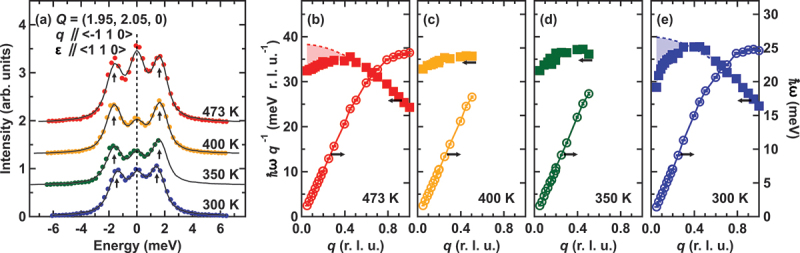
(a) Inelastic X-ray scattering spectra of the Fe-25Mn-3Mo single crystal sample measured at different temperatures: room temperature ∼300 K (blue), 350 K (green), 400 K (yellow) and 473 K (red). These measurements were conducted at the momentum transfer Q = (1.95, 2.05, 0) dQ = (−0.05, 0.05, 0) for the phonon propagation wave vector q∥⟨−110⟩, and the direction of the atomic displacement ε∥⟨110⟩. The solid lines represent the results of a fit using three Lorentzian components with resolution convolution. Solid lines serve as a visual guide. (b)–(e) reduced momentum transfer q dependence of phonon excitation energy divided by the energy transfer ($\hbar \omega q^{-1}$left axis) and phonon branch (ℏωright axis). Broken lines in (b) and (e) indicate the result of a fit with a simple sine curve.

Figure 3(a) depicts inelastic X-ray scattering spectra of the Fe-25Mn-3Mo single crystal measured at Q = (1.95, 2.05, 0) for the phonon propagation q∥⟨−110⟩, and the direction of the atomic displacement ε∥⟨110⟩. The spectra comprise three components: an elastic peak at ℏω=0 and two inelastic peaks, corresponding to Stokes and anti-Stokes peaks, corresponding to phonon creation and annihilation, respectively. The spectra were fitted using three Lorentzian’s functions convoluted with the resolution. The use of Lorentzians is reasonable when the intrinsic width of the phonon is much less than the energy, as was the case here. The fitting results are represented by solid lines, and the arrows indicate the positions of the phonon excitations. As observed in this figure, the phonon excitation energy at Q = (1.95, 2.05, 0) decreases 1.62 meV to 1.44 meV from 473 K to 300 K, indicating softening in the transverse phonon mode.

We investigate the dispersion of the TA mode to delve deeper into the details, we plotted the dispersion relation along the Γ to X point in Figure 3(b–e). For simple materials, phonon dispersion can be described by a sine curve:

(1) $$\hbar \omega =\sqrt{\frac{4C}{M}}\;|\sin\;\left(\frac{qa}{2}\right)|$$

where M, C, and a are the atomic mass, elastic constant, and lattice constant, respectively. According to this simple equation, the linear dispersion relation (ℏω∝q) is anticipated near the Γ point. While the observed dispersion is approximately fit by Eq. (1) across the entire q range, notable deviation from linear dispersion is observed as q→0. This deviation is evident near the Γ point as a distinct drop (shaded area in Figure 3(b,e) in the $\hbar \omega q^{-1}$ vs. q plot), becoming more pronounced in the long-wavelength limit at lower temperatures. Furthermore, we also examined whether anomalies in the long-wavelength limit of phonon dispersion could be associated with long-range structural phenomena. To explore the mesoscopic region, we conducted small-angle neutron scattering (SANS) experiments, but the SANS profiles showed no significant changes across the phase transition temperature. This suggests the absence of mesoscopic structural inhomogeneities.

Similar softening of $(C_{11}-C_{12})/2$ near the Γ point were reported in In-Tl [33], Nb-Ru [34], and Fe-Mn [23] alloys. A Nb-50Ru alloy undergoes martensitic transformation from a cubic B2 into a tetragonal L1${\;}_{0}$ phase, where the electron-phonon coupling was proposed as the origin of softening. Meanwhile, an Fe-30Mn system, which doesn’t show martensitic transformation, the contribution of local 3d electronic structure was suggested as an origin of Invar and phonon anomaly. Although several origins were proposed, mechanism of phonon anomaly is still under debate. Since the slope of the phonon dispersion relation (dℏω/dq) is proportional to the elastic constant $(C_{11}-C_{12})/2$, the observed softening indicates the presence of a bulk shear-mode instability in the parent fcc phase, even below $T_{\mathrm{Ms}}$. Moreover, this shear-mode instability persists not only below $T_{\mathrm{Ms}}$ but also above $T_{\mathrm{Mf}}$ in the parent phase, implying that the associated lattice strain energy is not fully relaxed by the partial martensitic transformation and the antiferromagnetic (AFM) phase transition.

It is noteworthy that the total energy of the γ→ε martensitic transformation system decreases with increasing hexagonality [35], suggesting that hexagonal local distortion may couple with the shear mode instability and contribute to phonon softening. In general, hexagonal distortion is associated with the elastic constant combination $(C_{11}-C_{12}+C_{44})/3$ and is coupled with the $\Gamma_{3}$ and $\Gamma_{5}$ phonon modes. However, it remains unclear whether both shear modes are involved in the elastic anomalies observed in FeMn-based Elinvar alloys. According to a previous study [23], no softening was observed in the $\Gamma_{5}$ phonon branch. Further investigations are therefore essential to fully understand the Elinvar characteristics of this alloy system.

We focused on the relationship between structural instability and the relaxation of lattice strain. This relationship has been studied in perovskite oxides undergoing displacement type structural transformations, such as (Ca,Sr)TiO${\;}_{3}$. In particular, phonon softening in SrTiO${\;}_{3}$ has been investigated using inelastic neutron and X-ray scattering techniques [36,37], revealing that the degree of softening depends on the Sr content. This compositional dependence modulates the internal lattice strain and leads to significant changes in material properties, including ferroelectricity [38].

Even though the order of the phase transition and the relevant phonon modes differ between perovskite oxides and FeMn alloys, these studies highlight the crucial role of lattice strain in structural instability in systems undergoing displacement type transformations. In the case of Fe-25Mn-3Mo, the parent phase remains partially untransformed even below the martensite start temperature, suggesting that the residual lattice strain and the stability of the parent phase are influenced by the Mn and Mo content. Although structural phase transitions typically lower the system’s energy, in Fe-25Mn-3Mo the martensitic transformation appears to be saturated, leaving behind a strained and unstable parent phase. We propose that this residual strain gives rise to phonon anomalies associated with structural instability, which persist over a wide temperature range across the transformation temperature. Based on this consideration, we suggest that a uniform local lattice distortion may be responsible for the phonon anomaly observed in the limit q→0. The results and discussion of local structure analysis will be presented in the next section.

### 3.3. Local atomic distortion

**Figure 4.**
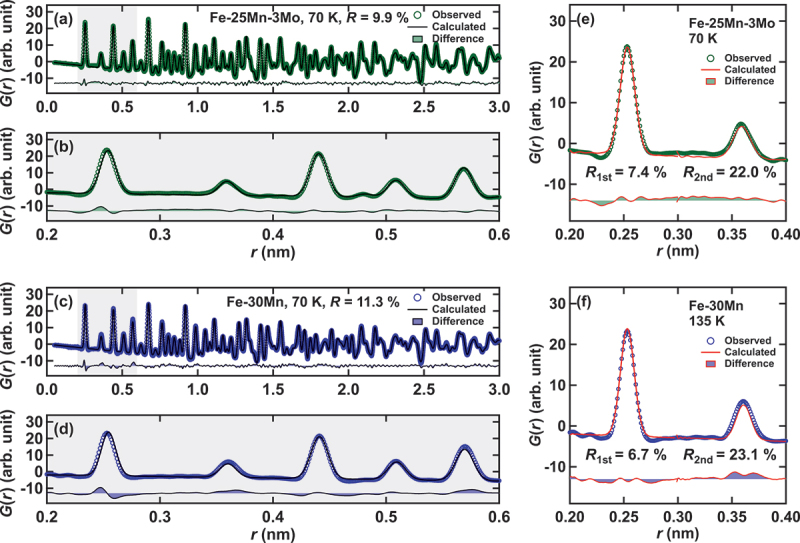
Reduced pair distribution function (denoted by circles), the result of a fit with a random structure model (solid lines), and the residuals between them for: (a, b) Fe-25Mn-3Mo and (c, d) Fe-30Mn. Subplots (b) and (d) provide magnified views of (a) and (c) from 0.2 to 0.6 nm, respectively. Subplots (e) and (f) display the results of the individual fitting method for Fe-25Mn-3Mo and Fe-30Mn, respectively, with red lines representing the curve fitting results.

We explored indications of lattice instabilities in atomic-scale local structures through neutron total scattering experiments using BL21 NOVA in MLF J-PARC for a super-Elinvar composition Fe-25Mn-3Mo and using BL-1B NOMAD in SNS ORNL for a reference composition Fe-30Mn. Figure 4 displays the reduced pair distribution function (PDF) of Fe-25Mn-3Mo and Fe-30Mn as a function of the real-space distance r. In this study, we utilized the reduced pair distribution function, defined as G(r)=4πrρ(g(r)−1), where ρ and g(r) represent the true density of the sample and the pair distribution function, respectively. It is important to note that in the reference alloy of Fe-30Mn, no Martensitic transformation is observed. Figures 4(a,b,e) and 4(c,d,f) present the G(r) for Fe-25Mn-3Mo and Fe-30Mn, respectively. In these figures, the open circles represent the observed G(r), the solid lines indicate the result of a fit, and the shaded areas depict the residual between them. For the entire r range dataset, the G(r) of Fe-25Mn-3Mo was fitted using a multi-phase model of the fully random Martensitic hcp and parent fcc phases, while that of Fe-30Mn was fitted with a simple random fcc model. Both models for the alloys appear to qualitatively reproduce the features of the profile across the entire r region.

**Figure 5.**
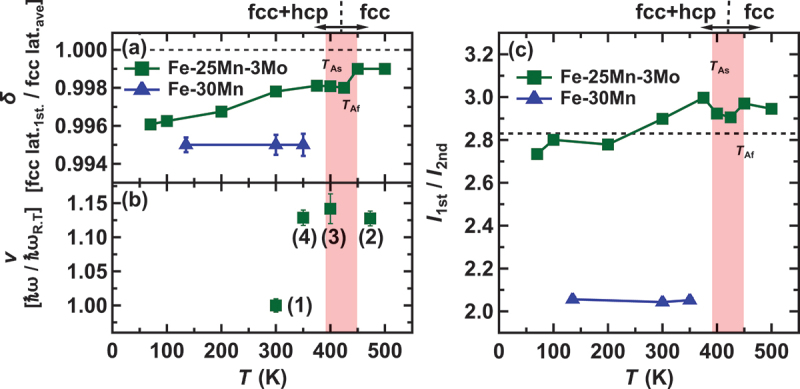
(a) Temperature dependence of the mismatch measure between local and global (averaged) structure for Fe-25Mn3Mo in heating process. Solid lines serve as a visual guide and all data points is including error bars (some bars are smaller than the point size). (b) Temperature dependence of the initial slope of the phonon dispersion normalized to room temperature, representing a normalized shear-mode elastic constant. (c) Temperature dependence of the intensity ratio between the first and second neighbors of G(r) in heating process. The dashed line represents the ideal intensity ratio for a fully random fcc/hcp structure. The solid lines serve as a visual guide for the data points is including error bar.

Upon closer examination, a significant difference in the peak position and width is evident at the first and second neighbors, as seen in the residuals in Figure 4, for both the super-Elinvar and reference compositions. This discrepancy may indicate deviations in atomic positions from the averaged structure. To quantify these deviations, we individually fitted the first and second peaks based on the following considerations. We first analyzed the full PDF profile (r=0∼3nm) using a dual-phase (fcc + hcp) model, optimizing both the volume fractions and lattice parameters of each phase. Subsequently, we analyzed only the first and second peaks, treating the lattice parameter of the fcc phase as the sole fitting parameter, and compared the results with those obtained from Rietveld refinement. The reason for using only the fcc lattice parameter in this local structure analysis is that the martensitic phase is metastable. It is reasonable to assume that the local structure of the hcp phase does not significantly deviate from its average structure, as any internal strain is likely relaxed through the structural phase transformation. In other words, we interpret the deviations between the experimental PDF profile and the one predicted from the average structure as arising from local atomic displacements in the fcc phase, which corresponds to the high-temperature parent phase. Figure 4(e–f) display the results of the fitting, indicating the boundaries of the range: (r=0.2∼0.3) and (r=0.3∼0.4) nm represent the fitting regions for the first and second peaks, respectively. As depicted in Figure 4(e–f), the individual fitting improves the reproducibility of the fitting. From these fitting results, we assessed the relative difference in the nearest neighbor distance between local and averaged structures. To quantitatively evaluate this relative difference, we suggest the parameter $\delta ,\delta =\left[(\mathrm{fcclat}.1\mathrm{st})/(\mathrm{fcclat}.\mathrm{ave})\right]$, where the numerator represents the local structure assessed from the G(r), and the denominator corresponds to the nearest-neighbor distance of the averaged structure evaluated from the Rietveld analysis. This relative difference δ serves as a measure of the mismatch between local and global (averaged) structures and is plotted as a function of temperature in Figure 5(a). The pair distribution function G(r) represents the distribution of atomic pairs at given relative distances. Even in a random alloy, we consider that the first-nearest-neighbor distance may deviate from the expected value in a perfectly periodic structure due to the influence of higher-neighbor distances, which smear the distribution of the first-nearest-neighbor distance. While this effect is challenging to visualize in real space, we believe Reverse Monte Carlo (RMC) calculations may provide a more detailed understanding. The measure of mismatch decreases with increasing temperature for Fe-25Mn-3Mo, while it remains almost independent of temperature for the reference fcc alloy Fe-30Mn. Although the size of δ for Fe-30Mn is larger than that for Fe-25Mn-3Mo, no Invar/Elinvar characteristics were observed.

It appears that the temperature dependence of the mismatch, (dδ/dT), rather than its absolute magnitude, plays a crucial role in the manifestation of Elinvar properties. Weiss proposed that the Invar effect arises from a volume change associated with transitions between the high-spin (HS) and low-spin (LS) states of iron [39]. This volume change, due to the HS – LS transition, has been experimentally and quantitatively verified in FeNi alloys [40]. A key feature of Weiss’s model is that the magnitude of the anomalous volume change is governed by the relative population of HS and LS states at a given temperature. Notably, the spin state of an Fe atom is influenced not only by temperature but also by its local environment. This implies that local structural and electronic inhomogeneities can significantly impact macroscopic thermal properties a concept that also applies to antiferromagnetic systems. More recently, the relationship between atomic pair distribution and local thermal expansion in FeNi alloys was investigated [41]. Their findings reveal a breakdown in the direct correlation between bulk thermal expansion and local anharmonic behavior. These results suggest that in random alloy systems, local lattice distortions due to atomic-scale displacements persist, and that the temperature dependence of the mismatch between local and average structures plays a significant role in macroscopic phenomena such as the Invar and Elinvar effects.

In this study, we observed a clear temperature dependence of the mismatch between average and local structures, particularly in the compositional and thermal regimes where the Elinvar effect emerges. In the case of Fe-25Mn-3Mo, the temperature evolution of this mismatch may indicate residual structural instability and appears to correlate with the observed phonon softening. Figure 5(b) shows the temperature variation of the initial slope of the phonon dispersion, defined as $v=(\hbar \omega /\hbar \omega_{RT})$, evaluated at |q|=0.05 and normalized to its value at room temperature. In general, Young’s modulus is approximately proportional to the second derivative of the interatomic potential U with respect to the atomic position R: $d^{2}U/dR^{2}$. Considering this, the mismatch between local and average structures may give rise to anomalous elastic properties through local variations in R that affect $d^{2}U/dR^{2}$. In contrast, the absence of temperature dependence in the mismatch observed for Fe-30Mn suggests that the local structure is stabilized within the fcc lattice framework, and that the structural instability is suppressed.

Based on our experimental result, we propose that slight local strains, distributed throughout the alloy, contribute to structural instability while preserving the long-range periodic structure. The identification of such small strains, even in a random alloy with only one independent atomic position, provides a new perspective for discussing elastic modulus anomalies. To further validate this proposal, theoretical calculations incorporating small local strains would be valuable. Further concrete interpretation of phonon softening and local structure in Invar/Elinvar alloys is worth investigating.

Finally, we address the potential short-range order in Fe-Mn alloys. In Figure 4, one can observe discrepancies in the heights of G(r) between the observed and fitted data, suggesting local distortion and/or compositional short-range ordering. Particularly in Figure 4(f), the fitting result for the second neighbor peak of Fe-30Mn appears subpar and slightly larger than that of the fitting curve. Since the height of G(r) is generally proportional to the number of atoms (coordination number), the ratio of the heights between the first and second neighbors should be independent of the concentration for fully random solid solutions. From a simple relationship between $G\left(r=r_{1st.}\right)$ and $G\left(r=r_{2nd.}\right)$, we can estimate the ideal ratio, *G*(*r* = *r*_1*st*._)/ *G*(*r* = *r*_2*nd*._) = [*N*_1*st*._/*r*_1*st*._] / [*N*_2*nd*._/*r*_2*nd*._] ∼ 2.83 for the fully random distribution. Here, $N_{i}$ and $r_{i}$ represent the i-th neighbor coordination number and the distance $\left(N_{1st.}=12,N_{2nd.}=6,r_{2nd.}=\sqrt{2}r_{1st.}\right)$, respectively. Comparing the observed and calculated ratio as shown in Figure 5(c), the ratio of Fe-25Mn-3Mo appears almost ideal, indicating a random distribution. Meanwhile, that of Fe-30Mn is significantly lower than the ideal value, implying atomic-scale compositional segregation. Following the principle of G(r), where the partial $G_{\mathrm{ij}}(r)$ of aniso-atomic pairs for Mn-Fe or Mn-Mo becomes negative, the positive residual at the second neighbor peak of Fe-30Mn (Figure 4(f)) indicates other pairs, such as Mn-Mn, Fe-Fe, Mo-Mo, and Fe-Mo, possibly favor. While the quantitative consistency of the short-range order parameters warrants examination through real-space structural modeling, the eutectic phase diagram of Fe-Mn appears to support atomic-scale phase separation.

## 4. Conclusion

In summary, our investigation into phonon dispersion and static local distortion via IXS and total-scattering experiments on the well-characterized super-Elinvar alloy Fe-25Mn-3Mo revealed significant findings. We observed a non-linear phonon dispersion anomaly for the transverse acoustic mode, associated with $(C_{11}-C_{12})/2$, across a wide temperature range. This anomaly is a clear indication of lattice instabilities coupled with tetragonal distortions in the long-wavelength limit. Our observations suggest that the interpretation of Elinvar characteristics based solely on the magnetostriction effect may be inadequate. Instead, we propose the existence of Martensitic transformation and parasitic lattice instabilities as underlying factors in these phenomena. Additionally, we highlight a significant mismatch between local and global (averaged) structures, indicating the influence of atomic-scale lattice instability in FeMn-based Elinvar alloys.
